# Evaluating the pharmacokinetics and safety of blonanserin tablets and Lonasen^®^: a randomized, open-label, two-period, two-sequence, self-crossover phase I clinical trial

**DOI:** 10.3389/fphar.2024.1511214

**Published:** 2025-01-03

**Authors:** Bo Qiu, Haojing Song, Xue Sun, Congyang Ding, Runxuan Du, Wanjun Bai, Zhanjun Dong

**Affiliations:** ^1^ Department of Pharmacy, Hebei General Hospital, Shijiazhuang, Hebei, China; ^2^ Hebei Key Laboratory of Clinical Pharmacy, Shijiazhuang, Hebei, China; ^3^ Department of Reproduction and Genetics, Hebei General Hospital, Shijiazhuang, Hebei, China

**Keywords:** blonanserin, schizophrenia, bioequivalence, pharmacokinetics, safety

## Abstract

**Objective:**

This study evaluated the pharmacokinetic and safety profiles of generic and original blonanserin tablets under fasting and postprandial conditions, and the bioequivalence of two formulations to obtain sufficient evidence for abbreviated new drug application.

**Methods:**

A randomized, open-label, two-period, two-sequence, self-crossover bioequivalence study was conducted to assess the bioequivalence of the test and reference blonanserin tablets under fasting and postprandial conditions. Eligible healthy individuals received a single 4-mg dose of either the test or reference blonanserin tablet, followed by a wash out period of 14 days. Serial blood samples were collected for up to 72 h after administration during each period, and the plasma concentrations of blonanserin were determined using a validated method. The non-compartmental method was used to calculate the primary pharmacokinetic parameters, and the geometric mean ratios for the PK parameters of the test drug to those of the reference drug, along with the corresponding 90% confidence intervals, were obtained for bioequivalence analysis. Throughout the study, a safety evaluation was conducted.

**Results:**

Under both fasting and postprandial conditions, the pharmacokinetic parameters of the test drug were found to be similar to those of the reference drug. The 90% confidence intervals (CIs) of the geometric mean ratios of the test to reference formulations were 97.79%–118.28% for peak concentration (C_max_), 92.35%–111.78% for the area under the curve from zero to the last measurable concentration (AUC_0-t_) and 92.88%–111.91% for the AUC from zero to observed infinity (AUC_0- ∞ _) under fasting conditions, 88.65%–103.20% for C_max_, 95.89%–106.81% for AUC_0-t_ and 96.02%–106.91% for AUC_0- ∞_ under postprandial conditions, all of which were within the accepted bioequivalence range of 80.00%–125.00%. Both the test and reference formulations were well-tolerated, and no serious adverse events related to the study drug were reported during the study.

**Conclusion:**

The bioequivalence of blonanserin tablets, both test and reference, was confirmed in healthy Chinese subjects under fasting and postprandial conditions, meeting the predetermined regulatory criteria for both formulations. Both formulations were found to be safe and well tolerated.

**Clinical Trial Registration:**

http://www.chinadrugtrials.org.cn/index.html, identifier CTR20230703.

## 1 Introduction

Schizophrenia is a prevalent, disabling mental disorder with significant social repercussions, the etiology and pathogenesis of which have yet to be fully explained (Owen et al., 2016). Approximately 21 million individuals across diverse age groups worldwide are affected by the condition. The disease is characterized by four distinct types of symptoms: positive, negative, emotional, and cognitive impairment (Fj et al., 2018). Patients typically require ongoing medication, which poses significant challenges for individuals, families, and society. Antipsychotic medications, commonly prescribed for schizophrenia, are generally categorized into two classes: conventional antipsychotics, such as chlorpromazine (first generation), and atypical antipsychotics, such as clozapine (second generation) (Leucht et al., 2009). Regrettably, a considerable portion of patients with schizophrenia, estimated to be one-third, do not respond to current treatments, as indicated by a recent survey (Menezes et al., 2006). Furthermore, both short-term and long-term clinical studies have demonstrated that the use of second-generation antipsychotic drugs may have harmful effects on various bodily systems, including the extrapyramidal motor system, glucose and lipid metabolism, the cardiovascular system, and sexual function (Moore and Furberg, 2017; Saito et al., 2022a; Saito et al., 2022b). Therefore, there is a pressing need to develop new medications that can enhance patient sensitivity to treatment while minimizing adverse effects.

Blonanserin is a relatively new antipsychotic medication with specific pharmacological mechanisms that was created in the early 1980s (Wu et al., 2022). This medication shows promise as a potential solution for unmet needs in the treatment of schizophrenia due to its selective action on specific receptors (Baba, et al., 2015). Blonanserin has been found to be an effective and well-tolerated treatment option for patients with schizophrenia, as it effectively addresses positive and negative symptoms, as well as cognitive symptoms, by binding to specific receptors such as 5-HT_2A_ serotonin, D_2_, and D_3_ dopamine receptors (Takaki et al., 2015). It has a low affinity for other neurotransmitter receptor subtypes, such as muscarinic M_1_, M_3_ receptors, 5-HT_2C_ serotonin receptors, histamine H_1_ receptors, and adrenoceptors α_1_ (Tateno et al., 2013). In short-term, multicenter, randomized controlled clinical trials (RCTs), blonanserin has demonstrated a lower risk of extrapyramidal symptoms (EPS) compared to haloperidol and is more effective in treating Positive and Negative Syndrome Scale (PANSS) negative symptoms (Harvey et al., 2019). Furthermore, blonanserin has been found to have a lower risk of hyperprolactinemia and weight gain than risperidone (Li et al., 2015).

The pharmacokinetic profile of blonanserin tablet (Lonasen^®^), originally developed by Sumitomo Dainippon Pharma Co.,Ltd., displays a T_max_ (time to reach C_max_) of 1.5 h, a T_1/2_ (terminal elimination half-life) of 10.7 h, and exhibits a higher potential for crossing the blood-brain barrier compared to Haloperidol, Risperidone, Olanzapine, and Sulpiride, resulting in a more significant brain-to-plasma concentration ratio (Deeks and Keating, 2010; Tateno et al., 2013). Blonanserin is primarily metabolized by the cytochrome P450 enzyme 3A4 in the liver and eliminated through urine, feces and bile (Deeks and Keating, 2010).

The efficacy and safety of Lonasen^®^ in patients with schizophrenia have been well established through extensive clinical trials (Kishi et al., 2013; Kishi et al., 2014). However, the high cost of the reference products places a financial burden on patients. Generic products, which usually have lower costs than original products, may provide a viable solution to alleviate patients’ financial burden and enhance the accessibility of antipsychotic medications. Furthermore, studies of blonanserin have been conducted in Japanese and Korean populations, and there is limited information available on PK studies of the Chinese population (Kishi et al., 2017; Jaewon et al., 2010). It is essential to evaluate the pharmacokinetic safety and tolerability of blonanserin in various racial and ethnic groups due to the differences in blonanserin levels observed among them.

Hebei Longhai Pharmaceutical Co., Ltd (China) recently developed a new generic blonanserin tablet. The National Medical Products Administration (NMPA) provided guidelines for this study, which assessed the pharmacokinetic (PK) characteristics of the test blonanserin tablet (T formulation) and compared them to those of the reference product (R formulation, Lonasen^®^). The purpose of this study was to support the marketing approval of the newly developed generic formulation in China. A bioequivalence study was performed in healthy Chinese participants under fasting and postprandial conditions. A generic drug is considered bioequivalent to the reference drug if the rate and extent of absorption of the two products are not significantly different. In addition, the pharmacokinetics and safety of blonanserin tablets in Chinese participants were explored.

## 2 Methods

### 2.1 Study drug

Blonanserin tablets (4 mg per tablet, batch number: 22,070,454), which were produced and provided by Hebei Longhai Pharmaceutical Co., Ltd., and blonanserin tablets (4 mg per tablet, batch number: 3036C), which are marketed under the brand name Lonasen^®^ and produced by Sumitomo Dainippon Pharma Co., Ltd., were used as T and R formulations, respectively, for the assessment of PK and BE studies.

### 2.2 Ethics approval and study population

The research was carried out at the Phase I Clinical Research Center of Hebei General Hospital from March to June 2023 and was registered with the Drug Clinical Trial Registration and Information Disclosure Platform [http://www.chinadrugtrials.org.cn/index.html] (number: CTR20230703, date: 13 Mar 2023). The study adhered to the NMPA’s Good Clinical Practice Guidelines, Declaration of Helsinki, and International Conference on Harmonization’s Good Clinical Practice Guidelines. The study protocol and any modifications were authorized by the Ethics Committee of Hebei General Hospital (approval No. 2023-02, 2023-03).

Eligible participants for the study were healthy men and women aged 18 or older with a body mass index of 19.0–26.0 kg/m2 (a minimum of 45 kg for females and 50 kg for males). These participants underwent a thorough medical examination, which included routine physical examination, measurement of vital signs, medical history, laboratory tests, 12-lead electrocardiography, and chest radiography, to assess their overall health and detect any clinically relevant diseases. To be eligible, the participants did not have any clinically significant abnormalities in the aforementioned examination items. Those with acute or chronic diseases not applicable to the study, history of alcohol or smoking abuse, drug abuse within the past year, allergy-prone constitution, or allergy to any ingredient in the blonanserin tablet were not eligible to participate. Additionally, individuals who had taken any medication within 28 days prior to the first dose, participated in any clinical trial within the past 3 months, suffered blood loss, or donated ≥200 mL of blood were excluded from the study Finally, pregnant or breastfeeding women and those likely to become pregnant were excluded from the study.

Before initiating the study, written informed consent was obtained from each participant, which contained information about the study’s objectives, procedures, and risk-benefit analysis. Additionally, participants were provided with the option to withdraw from the study at any point during the study period.

### 2.3 Study design

The present study was a single-center, randomized, open-label, two-cycle, two-sequence, self-crossover, phase I clinical trial featuring a 14-day washout period following each cycle. The trial comprised two independent trials: a fasting trial and a postprandial trial. The randomization process was performed using SAS statistical software (v9.4), which generated a random number table to allocate participants in the fasting trial into sequences A (TR) and B (RT) in a 1:1 ratio. Participants in the postprandial trial were randomly assigned to sequences C (TR) and D (RT) in a manner that mirrored the process of the fasting trial.

Each participant in the study received a single dose of either the T or R formulation, followed by oral administration of 240 mL of warm water. In the fasting trial, participants were required to fast for a minimum of 10 h prior to drug administration. In contrast, the postprandial trial involved consumption of a standard high-calorie, high-fat breakfast (800–1,000 kcal: protein, 150 kcal; carbohydrates, 250 kcal; fat, 500–600 kcal) 30 min before drug administration. During the study period, participants were not allowed to consume alcohol, engage in strenuous activities, or smoke. Additionally, a standardized meal was provided 4 and 10 h after treatment.

### 2.4 PK analysis

Blood samples were obtained using coded K_2_-EDTA anticoagulation tubes at specific time points in the fasting and postprandial trials. In fasting trial, samples were collected pre-dose at (0 h, baseline) and 0.25, 0.5, 0.75, 1, 1.25, 1.5, 1.75, 2, 2.5, 3, 4, 6, 8, 12, 24, 48, 72 h post-dose. In the postprandial trial, samples were collected pre-dose at (0 h, baseline) and 0.5, 0.75, 1, 1.25, 1.5, 2, 2.5, 3, 3.5, 4, 4.5, 5, 6, 8, 12, 24, 48, 72 h post-dose. After collection, the blood samples were centrifuged at 1,700 g for 10 min at 4°C within 1 h of collection. Plasma was then collected and stored at −70°C ± 10°C within 2 h of collection until analysis. Plasma samples were analyzed in a specialized analytical laboratory (Wuhan Hongren Biomedical Co., Ltd. Hubei, China), and concentrations of Blonanserin in plasma in healthy subjects were measured by a newly developed and validated LC-MS/MS method. LC-20ADXR (Shimadzu, JPN) and a Triple Quad 6500+Low Mass Spectrometer with ESI source (AB SCIEX, USA) were used. Briefly, blonanserin-d_5_ was added as internal standard, and protein was precipitated from the plasma samples. Then 10 μL of the supernatant was injected and separated on an Ultimate XB C18 (5.0 μm, 2.1 × 50.0 mm) column with gradient elution at a flow rate of 0.8 mL/min (mobile phase A, 0.1% acetic acid in water; mobile phase B, 0.1% acetic acid in acetonitrile). The column temperature was maintained at 40 °C. The multiple reaction monitoring (MRM) transitions of blonanserin and blonanserin-d_5_ were m/z 368.3/297.20 and m/z 373.30/297.20. The optimal instrument condition was set as follows: Heater temperature at 550°C; ion spray voltage as 5500 V; entrance potential was 10.0 V and collision cell exit potential was 13.0 V. Curtain gas was set as 40.0 psi, and gas 1 and gas 2 were both set as 55.0 psi. The linear range is 2.00 pg/mL to 2000.00 pg/mL for Blonanserin. The lower limits of quantitation (LLOQ) of Blonanserin was 2.00 pg/mL. The intra-batch and inter-batch precision in plasma samples were less than 7.2% and 5.3%. The intra-batch and inter-batch accuracy was in the range of −4.9% to 9.5% and −2.0%–4.4%.

The participants in the PK analysis set were selected from those who were randomly assigned to any group and completed periods 1-2 without significant deviations from the protocol. The primary PK endpoints for blonanserin were C_max_, AUC_0-t_, and AUC_0- ∞ _. The secondary endpoints included the terminal half-life of the analyte in the plasma (t_1/2_), time of maximum plasma concentration (T_max_), and terminal rate constant (λ_z_). The ABE criteria was used to evaluate the bioequivalence (BE) of blonanserin, with insignificant differences between the compared parameters (*p* > 0.05), and 90% confidence intervals (CI) for the geometric mean ratios (GMR) of C_max_, AUC_0-t_, and AUC_0- ∞_ falling within 80%–125% were deemed acceptable. In addition, the NMPA regulatory guidelines were followed by analyzing logarithm-transformed PK parameters using multivariate analysis of variance to determine the effects of the sequence, formulation, and period.

### 2.5 Safety analysis

Various laboratory tests, including biochemistry, hematology, and urinalysis, were performed to assess the safety of both formulations, along with monitoring of adverse events (AEs) and vital signs. Vital signs, including axillary temperature, blood pressure (systolic and diastolic), and pulse rate, were recorded 1 h before administration and at 2, 6, 12, 24, 48 and 72 h post-dose at each treatment visit. Laboratory and physical examinations, along with electrocardiography, were performed at baseline and 72 h after the second administration. AEs were coded according to the preferred terms and system organ classes in the Medical Dictionary for Regulatory Activities. The severity of adverse events was graded according to the National Cancer Institute Common Terminology Criteria for Adverse Events (NCI CTCAE 5.0), version 5.0. In addition to medical observations, the participants spontaneously reported any adverse events that occurred during the study.

### 2.6 Statistical analysis

Phoenix WinNonlin Software (Pharsight Corporation, version 8.3) was utilized to calculate pharmacokinetic (PK) parameters using the non-compartmental method, and individual plasma concentration-time curves were constructed. Following logarithmic transformation, the primary PK parameters derived from the plasma concentration-time curve were subjected to multivariate analysis of variance, which included participants nested within the sequence as a random effect and sequence, formulation, and period as fixed effects. Two-sided t-tests were used for statistical analysis, and Tmax was analyzed using the non-parametric Wilcoxon two-sample test. Descriptive statistics were used for PK parameters, and count or grade data were expressed as frequencies and percentages. Statistical analyses were performed using the SAS software package version 9.4, with statistical significance set at *p* < 0.05.

## 3 Results

### 3.1 Participant demographics

**FIGURE 1:**
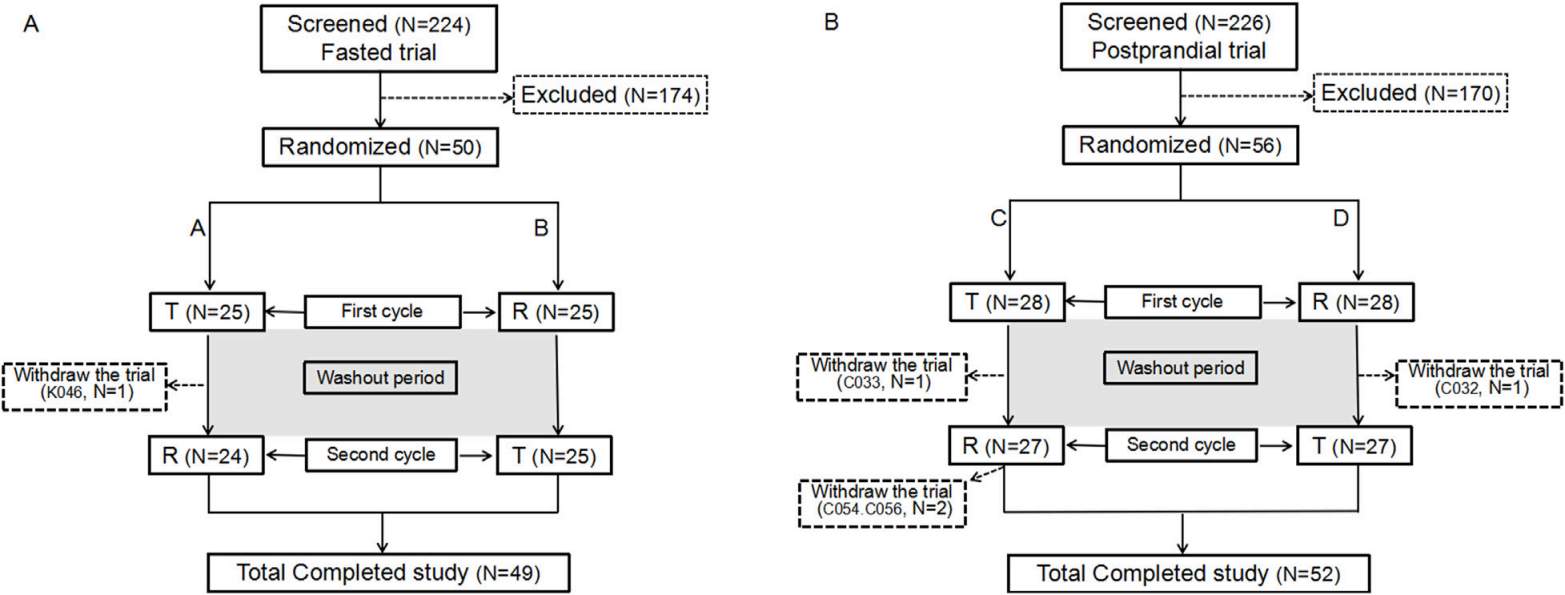
Participant flow chart. Flow chart of the participants in the fasting state **(A)**. Flow chart of the participants in the postprandial state **(B)**. N: the number of participants.

The trial screening process for fasting and postprandial conditions enrolled 224 and 226 participants, respectively. Of these, 50 and 56 were enrolled in the fasting and postprandial trials, respectively. In the fasting group, 9 women and 41 men were enrolled, whereas in the postprandial group, 15 women and 41 men were enrolled. One participant in the fasting group and four in the postprandial group withdrew from the trial. Figure 1 illustrates the screening and inclusion distributions of the participants in the two groups.

In the fasting group, participant K046, who had completed the initial cycle of blood collection, opted to withdraw from the study for personal reasons and did not receive the R formulation in the second cycle. According to the principles of trial completion, dropout, protocol deviation, exclusion, and statistical analysis, 50 participants were enrolled in the full analysis set (FAS), safety analysis set (SS), pharmacokinetic parameter analysis set (PKPS), pharmacokinetic concentration analysis set (PKCS), and bioequivalence analysis set (BES). No adverse events in the fasting group led to withdrawal from the study.

In the postprandial group, 56 participants were assigned to the FAS, PKPS, PKCS, SS, or BES sets. Four participants (C032, C033, C054, and C056) discontinued participation in the trial. Participants C032 and C033 completed the first cycle and withdrew from the trial before the second cycle. C054 withdrew for personal reasons following the blood collection process within 24 h of the second cycle. C056, on the other hand, withdrew due to the occurrence of an adverse event (upper respiratory tract infection) 12 h after the commencement of the second cycle.

The baseline characteristics of each participant are detailed in Table 1. It is noteworthy that all participants fulfilled the inclusion criteria established for the study.

**TABLE 1:** Demographic baseline.

Variable	Fasting trial	Postprandial trial
Age (years)		
Mean ± SD	30.1 ± 8.30	34.2 ± 9.48
Median (Q1; Q3)	29.0 (23.0,37.0)	34.5 (25.0,42.0)
Min; Max	19,49	18,51
Sex n (%)		
Male	41 (82.0%)	41 (73.2%)
Female	9 (18.0%)	15 (26.8%)
Ethnicity n (%)		
ethnic Han	50 (100%)	53 (94.6%)
Others	0	3 (5.4%)
Height (cm)		
Mean ± SD	169.04 ± 6.402	166.85 ± 7.490
Median (Q1; Q3)	170.00 (164.50,173.50)	168.25 (161.25,172.75)
Min; Max	149.0.182.5	152.5,182.0
Weight (kg)		
Mean (Std)	65.02 ± 8.244	64.21 ± 8.548
Median (Q1; Q3)	62.70 (58.00,73.00)	64.90 (57.10,70.85)
Min; Max	52.0,80.3	46.9,82.8
BMI (kg/m2)		
Mean (Std)	22.69 ± 1.960	22.98 ± 1.824
Median (Q1; Q3)	22.60 (20.90,24.30)	23.30 (21.50,24.35)
Min; Max	19.4,25.9	19.4,26.0

### 3.2 PK results

**FIGURE 2:**
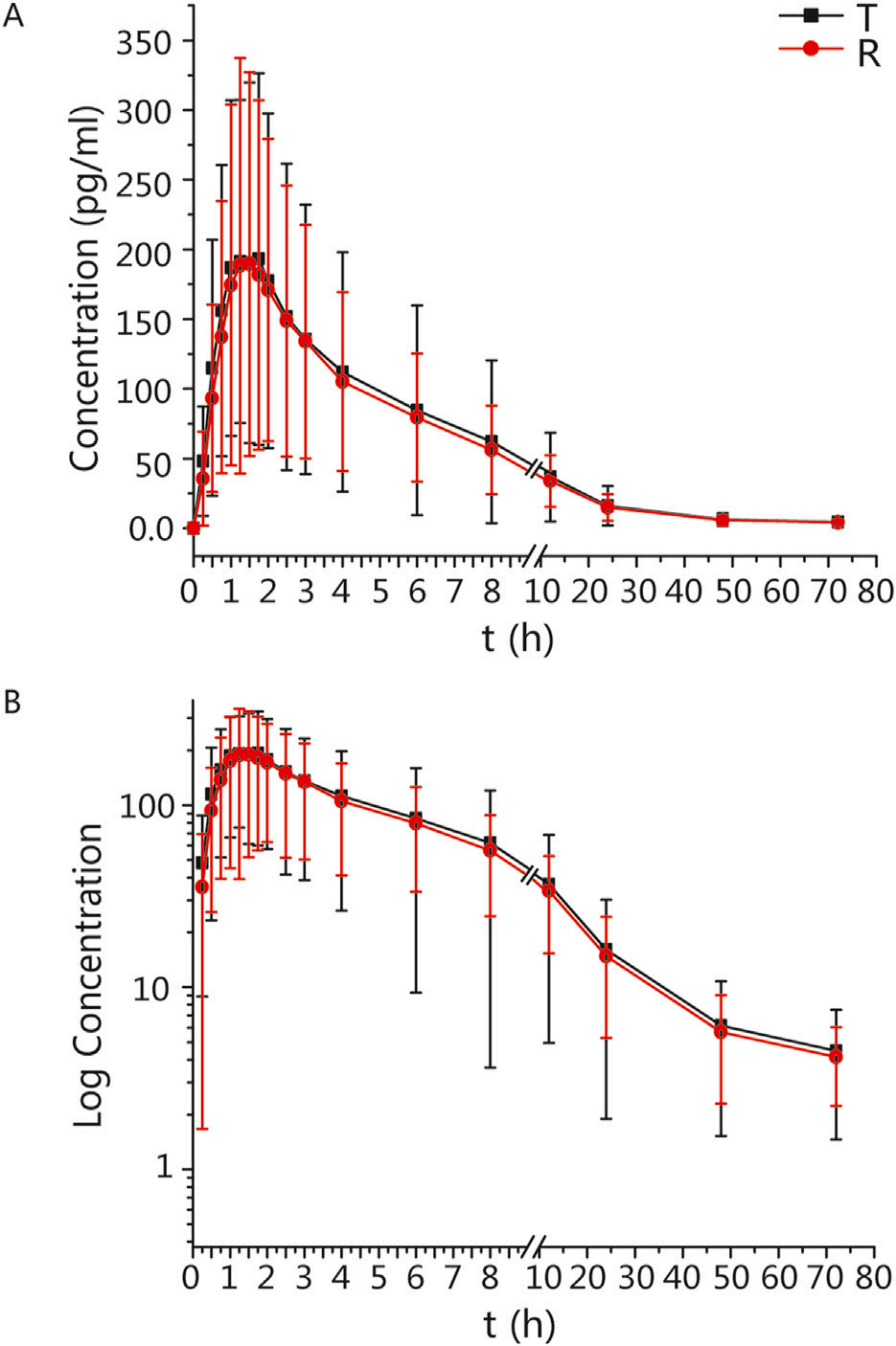
PK analysis of blonanserin of T and R formulations. Mean blood concentration (±SD) time curve after oral T and R formulations during fasting: arithmetic mean **(A)** and log transformation **(B)**.

**FIGURE 3:**
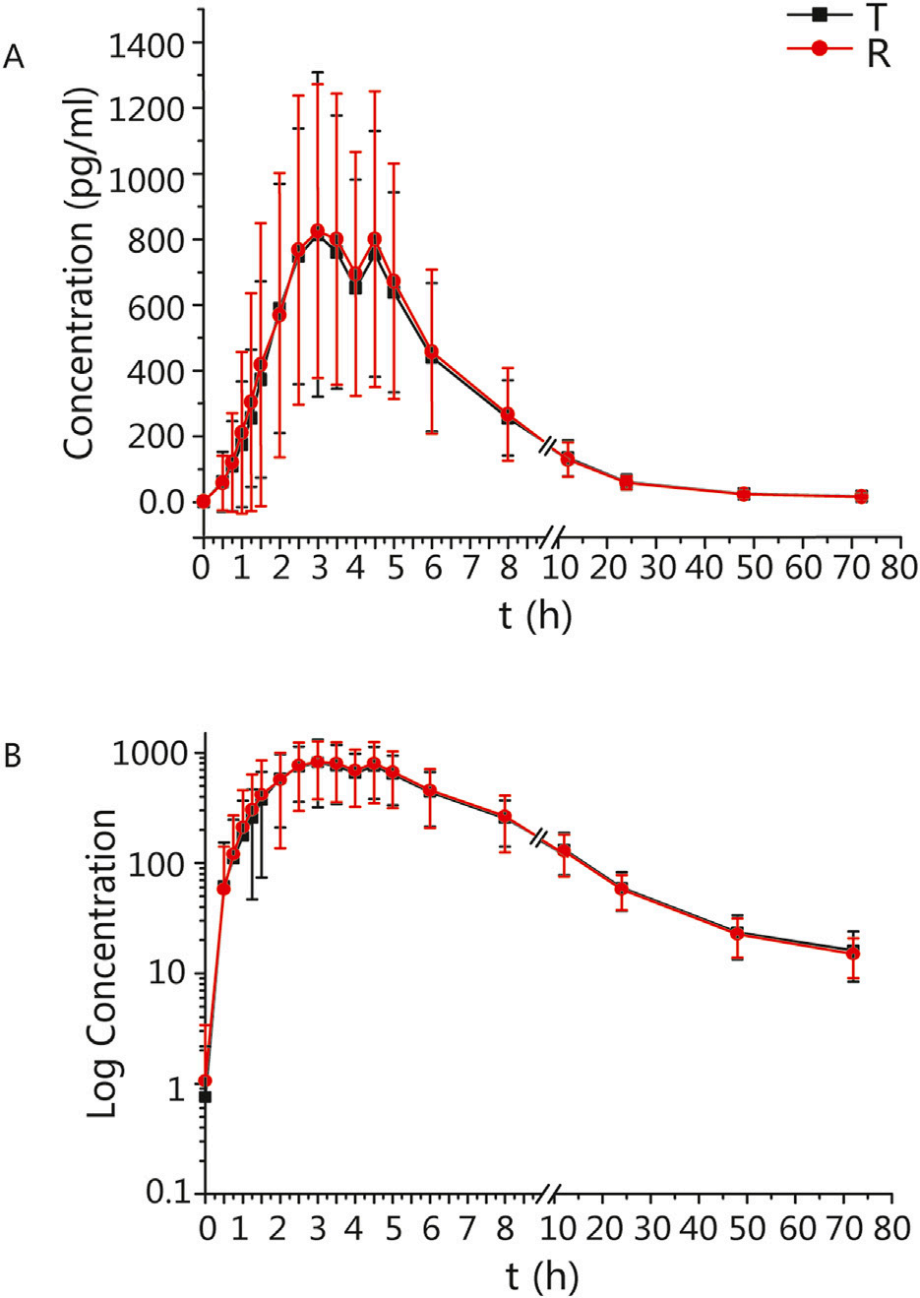
PK analysis of blonanserin of T and R formulations. Mean blood concentration (±SD) time curve after oral T and R formulations during postprandial: arithmetic mean **(A)** and log transformation **(B)**.

The mean ± standard deviation (SD) plasma drug concentration-time curves for blonanserin under fasting and postprandial conditions after completing the two cycles are shown in Figures 2, 3 respectively.

In the fasting group, the main PK parameters for blonanserin of the T and R formulations were as follows: C_max_, AUC_0–t_, and AUC_0– ∞_ values were 234.39 ± 150.51 pg/mL and 216.33 ± 151.94 pg/mL, 1784.25 ± 1,331.17 h*pg/mL and 1,663.85 ± 995.27 h*pg/mL, and 1890.64 ± 1,402.93 h*pg/mL and 1759.95 ± 1,045.82 h*ng/mL, respectively.

In the postprandial group, the main PK parameters for blonanserin of the T and R formulations were as follows: The C_max_, AUC_0–t_, and AUC_0– ∞_ values were 992.10 ± 479.62 pg/mL and 1,031.60 ± 490.81 pg/mL, 7,258.58 ± 2,732.82 h*pg/mL and 7,247.83 ± 2,755.25 h*pg/mL, 7,796.34 ± 3,037.15 h*pg/mL and 7,763.66 ± 2,959.70 h*pg/mL, respectively. The detailed PK parameters are shown in Table 2.

**TABLE 2:** The PK parameters of blonanserin after oral T and R formulations under fasting and postprandial conditions.

PK parameters	Fasting trial		Postprandial trial	
	Mean ± SD		Mean ± SD	
	T	R	T	R
T_max_(h)	1.25 (0.50–6.00) (N = 50)	1.25 (0.50–3.00) (N = 49)	3.00 (1.00–8.00) (N = 55)	3.00 (1.00–5.00) (N = 55)
C_max_ (pg/mL)	234.39 ± 150.51 (N = 50)	216.33 ± 151.94 (N = 49)	992.10 ± 479.62 (N = 55)	1,031.60 ± 490.81 (N = 55)
AUC_0-t_ (h·pg/mL)	1784.25 ± 1,331.17 (N = 50)	1,663.85 ± 995.27 (N = 49)	7,258.58 ± 2,732.82 (N = 55)	7,247.83 ± 2,755.25 (N = 55)
AUC_0- ∞ _(h·pg/mL)	1890.64 ± 1,402.93 (N = 50)	1759.95 ± 1,045.83 (N = 49)	7,796.34 ± 3,037.15 (N = 55)	7,763.66 ± 2,959.70 (N = 55)
λz (h^-1^)	0.046 ± 0.016 (N = 50)	0.047 ± 0.017 (N = 49)	0.032 ± 0.006 (N = 55)	0.037 ± 0.030 (N = 55)
t_1/2_(h)	16.97 ± 5.85 (N = 50)	16.58 ± 5.619 (N = 49)	22.36 ± 4.76 (N = 55)	21.84 ± 5.99 (N = 55)

To determine if period, sequence, and formulation factors affected bioequivalence in fasting and postprandial states, we conducted a linear mixed model analysis and multivariate analysis of variance for each primary parameter (Table 3). Our results showed that the mixed-effects model did not have a significant impact on the conclusions of the study in either state, with *p* > 0.05 for both fasting and postprandial states.

**TABLE 3:** The results of variance analysis of the main PK parameters after logarithmic transformation.

Effect factor	Fasting trial (*P*)			Postprandial trial (*P*)		
	Ln (C_max_) (pg/mL)	Ln (AUC_0-t_) (h*pg/mL)	Ln (AUC_0- ∞ _) (h*pg/mL)	Ln (C_max_) (pg/mL)	Ln (AUC_0-t_) (h*pg/mL)	Ln (AUC_0- ∞ _) (h*pg/mL)
Sequence	0.4298	0.8468	0.8673	0.3107	0.2784	0.2918
Period	0.0547	0.0295	0.0208	0.0639	0.2422	0.2017
Formulation	0.2053	0.7815	0.7293	0.3315	0.7113	0.6842

### 3.3 BE results

In fasting trials, the ABE criterion was adopted to evaluate the bioequivalence of the PK parameters. The GMR of C_max_ for blonanserin was 107.55%, and the 90% CI was 97.79%–118.28%. The GMR of the AUC_0–t_ for blonanserin was 101.60%, and the 90% CI was 92.35%–111.78%. The GMR of the AUC_0– ∞_ for blonanserin was 101.95%, and the 90% CI was 92.88%–111.91% (Table 4). ABE criteria was adopted for the postprandial trial. The GMR of the C_max_ for blonanserin was 95.65%, and the 90% CI was 88.65%–103.20%. The GMR of the AUC_0–t_ for blonanserin was 101.21%, and the 90% CI was 98.89%–106.81%. The GMR of the AUC_0– ∞_ for blonanserin was 101.32%, and the 90% CI was 96.02%–106.91% (Table 5).

**TABLE 4:** Results of the equivalence determination of the T and R formulations in the fasting trial.

Bioequivalence determination of blonanserin									
PK parameters	T		R		GMR (%)	CV_WR_ (%)	90%CI	Power (%)	Bioequivalence Criteria met?
	N	GM	N	GM					
C_max_ (pg/mL)	50	196.27	49	182.49	107.55	28.64	97.79–118.28	83.4	YES
AUC_0-t_ (h*pg/mL)	50	1,464.60	49	1,441.54	101.60	28.76	92.35–111.78	96.8	YES
AUC_0- ∞ _ (h*pg/mL)	50	1,559.57	49	1,529.72	101.95	28.03	92.88–111.91	97.2	YES

**TABLE 5:** Results of the equivalence determination of the T and R formulations in the postprandial trial.

Bioequivalence determination of blonanserin									
PK parameters	T		R		GMR (%)	CV_WR_ (%)	90%CI	Power (%)	Bioequivalence Criteria met?
	N	GM	N	GM					
C_max_ (pg/mL)	55	898.25	55	939.11	95.65	23.97	88.65–103.20	98.7	YES
AUC_0-t_ (h*pg/mL)	55	6867.32	55	6785.54	101.21	16.88	95.89–106.81	>99.9	YES
AUC_0- ∞ _ (h*pg/mL)	55	7,359.45	55	7,263.54	101.32	16.81	96.02–106.91	>99.9	YES

**FIGURE 4:**
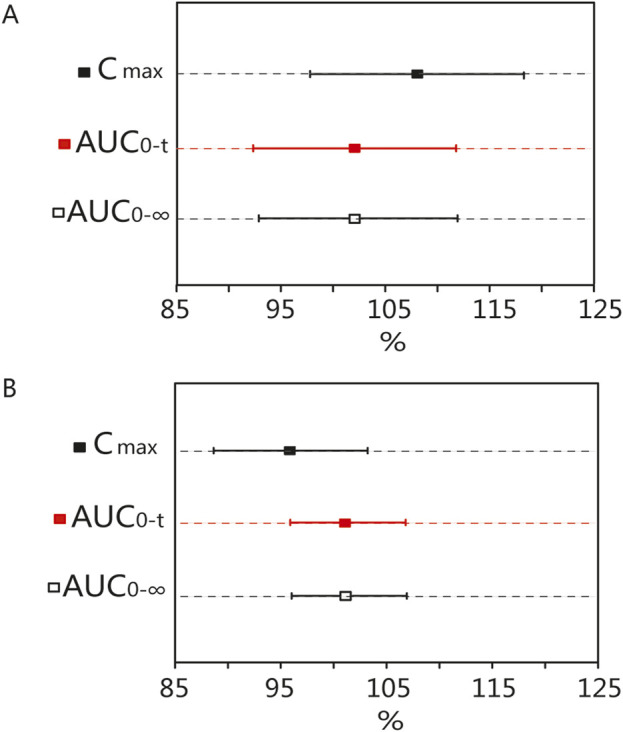
Bioequivalence analysis of T and R formulations. The bioequivalence analysis of blonanserin of T and R formulations during fasting **(A)** shows the ratio range of C_max_, AUC_0-t_ and AUC_0– ∞_ of T and R formulations with 90% CIs. The bioequivalence analysis of blonanserin of T and R formulations during postprandial **(B)** shows the ratio range of C_max_, AUC_0-t_ and AUC_0– ∞_ of T and R formulations with 90% CIs. (bioequivalence was declared if the 90% CIs were within the prespecified acceptable ranges of 80%–125%). C_max_: the maximum observed drug concentration in the plasma; AUC_0– ∞ _: AUC of the analyte in the plasma over the time interval from time zero to infinity; AUC_0-t_: AUC of the analyte in the plasma over the time interval from time zero to the last measurable concentration.

According to the data, all primary pharmacokinetic (PK) parameters had 90% confidence intervals (CIs) that fell within the range of 80.00%–125.00% (Figure 4). This indicates that the criteria for bioequivalence were met. Additionally, the PK parameters listed in Table 2 further supported the conclusion that the PK profiles of the T and R formulations were comparable, confirming their bioequivalence.

### 3.4 Safety results

Both formulations exhibited good safety in healthy Chinese participants in both fasting and postprandial states. Seventeen AEs were reported in nine participants in the test product; the incidence of AEs was 18% (9/50), whereas 11 AEs were reported in seven participants and an incidence of 14.3% (7/49) in the reference product. The incidence of AEs in the fasting group was 32.0%; 28 AEs occurred in 16 participants, all of which were mild and reported as grade 1 or 2, except for one case of AE reported as grade 3, which was judged to be possibly related to the study drug. The incidence of AEs in the fasting group is shown in Table 6.

**TABLE 6:** Summary of AEs in the fasting trial.

Parameters	T (N = 50)			R (N = 49)		
	E	n	%	E	n	%
sum	17	9	18.0	11	7	14.3
AE severity						
Grade 1	13	6	12.0	11	7	14.3
≥Grade 2	4	3	6.0	0	0	0
Correlation with drugs						
Possible related	9	7	14.0	8	5	10.2
Possible unrelated	8	5	10.0	3	2	4.0
Influenza	1	1	2.0	0	0	0
Leukocytes count decreased	1	1	2.0	0	0	0
ALT increased	0	0	0	1	1	2.0
Lymphocytes % decreased	0	0	0	1	1	2.0
Positive urine leukocytes	0	0	0	2	1	2.0
Urine sediment detected	0	0	0	1	1	2.0
Positive urine erythrocyte	2	2	4.0	0	0	0
Positive urine occult blood	2	2	4.0	0	0	0
Eosinophil % increased	2	2	4.0	0	0	0
Eosinophil count increased	1	1	2.0	0	0	0
AST increased	1	1	2.0	2	2	4.0
ECG abnormality	1	1	2.0	0	0	0
ECG abnormality	3	3	6.0	2	2	4.0
Hyperkalemia	0	0	0	1	1	2.0
Blood pressure increased	1	1	2.0	0	0	0
Neutrophil count decreased	2	2	4.0	0	0	0
Sinus bradycardia	0	0	0	1	1	2.0

Abbreviations: AST, aspartate aminotransferase; ALT, alanine aminotransferase; ECG, electrocardiogram.

The incidence of AEs in the postprandial group was 67.9%, with 58 AEs occurring in 38 participants. 57 AEs were considered mild and did not require any further treatment. One AE (right calcaneal fracture) was reported as grade 3 in severity and judged to be unrelated to the study drug. The AEs in the postprandial group are summarized in Table 7. All AEs resolved or remained stable even after the end of the study, and the results showed that the incidence and types of AEs were similar between the two formulations under fasting and postprandial conditions.

**TABLE 7:** Summary of AEs in the postprandial trial.

Parameters	T (N = 55)			R (N = 55)		
	E	n	%	E	n	%
sum	28	19	34.5	30	19	34.5
AE severity						
Grade 1	28	19	34.5	29	18	32.7
≥Grade 2	0	0	0	1	1	1.8
Correlation with drugs						
Possible related	9	5	9.1	16	11	20.0
Possible unrelated	19	18	32.7	13	10	18.2
Definitely unrelated	0	0	0.0	1	1	1.8
ALT increased	2	2	3.6	1	1	1.8
AST increased	3	3	5.5	0	0	0.0
TG increased	2	2	3.6	3	3	5.5
Creatinine increased	2	2	3.6	0	0	0
Blood glucose decreased	8	8	14.5	3	3	5.5
Blood glucose increased	1	1	1.8	0	0	0
Blood uric acid increased	2	2	3.6	0	0	0
Fibrinogen content increased	1	1	1.8	0	0	0
ECG abnormality	3	3	5.5	2	2	3.6
Hypophosphoria	3	3	5.5	5	5	9.1
Total cholesterol increased	1	1	1.8	1	1	1.8
Lymphocytes % decreased	0	0	0	1	1	1.8
Haemoglobin decreased	0	0	0	3	3	5.5
Upper respiratory infection	0	0	0	1	1	1.8
Eosinophils increased	0	0	0	2	2	3.6
Fibrinogen content decreased	0	0	0	1	1	1.8
Platelet count increased	0	0	0	1	1	1.8
Right calcaneus Fracture	0	0	0	1	1	1.8
Blood bilirubin increased	0	0	0	4	4	7.2

Abbreviations: AST, aspartate aminotransferase; ALT, alanine aminotransferase; TG, triglyceride; ECG, electrocardiogram.

## 4 Discussion

According to the Chinese legal requirements, if the difference in absorption rate and degree between the T and R formulations falls within the prescribed acceptable range, the T formulation can be utilized for marketing application. Blonanserin is a compound with a relatively simple molecular structure and straightforward metabolic pathway *in vivo*. Upon demonstrating the consistency of pharmacokinetic parameters between the T and R formulations, it can be inferred that the two formulations exhibit therapeutic equivalent. To minimize drug exposure, we conducted a two-period crossover study involving 50 and 56 participants. Consequently, owing to sample size constraints and single-dose administration, a comprehensive evaluation of the efficacy and safety profiles of the two drugs is hindered. The long-term efficacy and safety of the T formulation can only be evaluated through a postmarketing real-world study. If the efficacy and safety of the investigational product prove consistent with those of the reference product, the lower cost economic value of the investigational product post-marketing would be substantial.

In this study, the process of drug administration and blood collection was undertaken. Given that the absorption, distribution, and elimination phases of blonanserin must be considered for during the sample period, it is essential to consider the time-point design in our study. Additionally, we examined the PK data from both fasting and postprandial trials and discovered that the primary PK parameters of blonanserin in the postprandial condition were notably higher than those in the fasting condition. It is hypothesized that the pharmacokinetic parameters of blonanserin, which possesses greater lipid solubility, are significantly influenced by the consumption of high-fat diets. The chemical name of blonanserin is 2-(4-Ethyl-1-piperazinyl)-4-(4-fluorophenyl)-5,6,7,8,9,10-hexahydrocycloocta [b]pyridine (Deeks and Keating, 2010). In the acidic environment of the stomach (pH 1–6) and intestine (pH 5–8) (Guerra et al., 2012; Sjgren et al., 2014) blonanserin, with a pKa value of 7.7, is ionized by at least 50% (Dening et al., 2018). This makes it difficult for the ionized blonanserin molecules to be absorbed throughout the gastrointestinal tract. However, food elevates intragastric pH and decreases the rate of gastric emptying, which results in increased solubility of blonanserin, thereby increasing drug absorption (Saruwatari et al., 2010). Our study revealed a substantial increase in the uptake of blonanserin during the postprandial period, a finding that is consistent with the outcomes of other investigations (Shang et al., 2018). Based on our observations, it appears that hepatoenteral circulation was present in the postprandial group, but not in the fasting group. Prior studies have indicated that bile secretion is a critical pathway for blonanserin clearance *in vivo* (Shang et al., 2018). Dietary fat content can significantly impact bile secretion and excretion, with high-fat foods leading to increased bile secretion and excretion, while carbohydrate foods have a lesser impact (Singh, 1999). As a result, after consuming a high-fat diet, more blonanserin is excreted into the duodenum through the bile, leading to secondary re-absorption, which can explain the double peak in the postprandial blood concentration curve.

Blonanserin is a substrate of CYP3A4 and its exposure *in vivo* can be affected by inhibitors of CYP3A4, as it is a substrate of this enzyme (Deeks and Keating, 2010). Therefore, any factors that influence the activity of CYP3A4 could potentially impact exposure to blonanserin. By analyzing the co-administration of grapefruit juice with 8 mg of blonanserin, Shang et al. revealed that the systemic exposure to blonanserin significantly increased when grapefruit juice was absorbed into the circulation alongside the drug (Shang et al., 2018). Moreover, CYP3A4 inhibitors, CYP3A4 inducers, central nervous system depressant drugs, and dopamine agonists can also influence the bioavailability of blonanserin (Shang et al., 2018). Considering the impact of variable bioavailability on efficacy and toxicity, it is necessary to adjust the dose of blonanserin for each patient or population based on the intake of food or drugs that may affect its bioavailability. As schizophrenia is a chronic disease that requires long-term medication, it is crucial to consider the potential effects of food and concomitant medication on blonanserin bioavailability and adjust the dose accordingly (Hasan et al., 2013).

Despite demonstrating promising outcomes in our clinical trials, some limitations have been identified. While the sample size of this study meets the requirements for statistical analysis of bioequivalence, it imposes limitations on the evaluation of safety. The findings of this study can demonstrate pharmacokinetic equivalence between the two drugs, and inferred indirectly that the two formulations exhibit therapeutic equivalent. Consequently, future investigations with larger sample sizes will be necessary to corroborate the conclusions drawn from this study. The data were collected from healthy Chinese participant population, and our results indicated that the exposure of blonanserin in the Chinese population was significantly higher than that in the Japanese population in both fasting and postprandial states. Furthermore, there is a lack of published data regarding the pharmacokinetic parameters of blonanserin in other ethnic populations, consequently, caution should be exercised when interpreting the results, as they may not be generalizable to patients of all races who are more likely to use the drug.

## 5 Conclusion

The formulation of blonanserin tablets in the T configuration was equivalent to the R formulation tablet under both fasting and postprandial conditions, meeting the criterion for bioequivalence. The effect of food on the absorption of blonanserin was observed to be significant. Both formulations were safe and well-tolerated. This study lays the groundwork for future clinical trials of blonanserin tablets and paves the way for clinical application of blonanserin tablets in China.

## Data Availability

The original contributions presented in the study are included in the article/supplementary material, further inquiries can be directed to the corresponding authors.
